# Causes of admission and outcomes of brown hare (*Lepus europaeus*) leverets at wildlife rescue centres in the Czech Republic

**DOI:** 10.1186/s12917-021-03136-w

**Published:** 2022-01-15

**Authors:** Gabriela Lukesova, Eva Voslarova, Vladimir Vecerek, Katarina Nenadovic

**Affiliations:** 1 Department of Animal Protection and Welfare and Veterinary Public Health, Faculty of Veterinary Hygiene and Ecology, University of Veterinary Sciences Brno, Brno, Czech Republic; 2 Department of Animal Hygiene, Faculty of Veterinary Medicine, University of Belgrade, Bulevar oslobodjenja 18, Belgrade, 11000 Serbia

**Keywords:** Mammal, Orphan, Rehabilitation, Mortality, Release

## Abstract

**Background:**

Wildlife rescue centres care for orphaned and injured young as an integral part of their work. However, inappropriate interventions in nature can have a negative effect on the survival of young hares, especially when the care of these young is not very successful. The aim of this study was to assess the number of brown hare leverets admitted to rescue centres in the Czech Republic in the period from 2010 to 2019, the causes of their admission to rescue centres and their outcomes.

**Results:**

We evaluated the number of brown hare leverets admitted to rescue centres in the Czech Republic in the period from 2010 to 2019 and the outcomes associated with their leaving these rescue centres. We found that the number of brown hare leverets admitted increased during the monitored period (rSp = 0.6364, *p* < 0.05). The most frequent reasons for admission were the admission of orphaned young (49.15%), leverets brought needlessly (19.60%) and leverets that had been bitten by other animals (18.63%). More (p *<* 0.05) young admitted to rescue centres died (40.76%) than were reared successfully and released back into the wild (32.40%). Leverets that had been caught needlessly or orphaned and late-born leverets survived and could be released back into the wild (38.56, 34.51 and 52%, respectively), while fatalities were recorded in most leverets bitten by another animal (65.05%) or hit in a collision with a vehicle (97.06%). Most young hares (76.92%) that were exhausted or starved at the time of admission could not be saved.

**Conclusions:**

Since only a small proportion of hares in a litter survive until adulthood in the wild, young animals being found and taken needlessly to rescue centres may harm the hare population. Our results show that only around one in three healthy young hares admitted to rescue centres are reared successfully. It is, in our opinion, of fundamental importance to the protection of brown hare leverets to inform the public of this issue and prevent needless interventions into natural rearing in the wild.

## Introduction

The brown hare (*Lepus europaeus*) is a common species occurring in the Czech Republic and other European countries [1]. The degree of the threat to this species stated by the International Union for Conservation of Nature (2020) ranges from Least Concern to Near Threatened depending on the area in which it occurs. Hare populations are relatively isolated as a result of the mosaic distribution of the landscape divided by large roads, train tracks and towns [2]. Their natural habitat is in decline or significantly transformed by the extensive use of land for agriculture, which is reflected in a decline in the numbers of these animals [3, 4]. Furthermore, hares are often forced to cross roads when seeking new habitats, which leads to collisions with vehicles [5]. Several diseases and infections of parasitic origin [6] and natural predators [7] also play a large part in the decline in hare numbers, in addition to anthropogenic activity.

Although hares develop a large number of survival strategies [8], their mortality rate is extremely high, particularly among young in the first two weeks of life [9]. Hansen [10] reported a mortality rate of as much as 80% in leverets as a result of a shortage of suitable habitats. In their study, Karp and Gehr [11] reported an even lower survival rate in young brown hares, likewise due to the unsuitability of habitats. Litters are also affected negatively by excessive precipitation [12] and the occurrence of large numbers of parasites that cause a decline in the weight and hardiness of animals [6]. The mortality rate is also high among adult hares, which are affected by poaching, predation and the aforementioned collisions with road traffic [13]. Because this is a game animal, gamekeeping and other hunting activities also make their mark on hare populations [14, 15].

Caring for hares is far from easy despite developments made in veterinary medicine. Hares are animals that are extremely stress-sensitive, which has an impact on their health and results in high mortality [16], and for this reason, they have specific handling requirements [17]. Young hares can get in a negative energy balance as a result of stress and the refusal of food, which harms their health [18]. Rescue centres contribute to nature protection by helping to restore handicapped animals to health and return them to the wild, thereby compensating in part for the negative effects of anthropogenic activities on these animals.

This study aimed to assess the number of leverets admitted to rescue centres in the Czech Republic in the period from 2010 to 2019 and to assess the causes of their admission to rescue centres and their outcomes.

## Materials and methods

We obtained data on handicapped animals admitted to rescue centres in the Czech Republic from the records of the Ministry of the Environment, which coordinates the activity of rescue centres. The subject of our evaluation was all brown hare leverets admitted to the rescue centres falling under the National Network of Rescue Centres of the Czech Republic in the period from 2010 to 2019, as well as the reasons for their admission and their outcomes.

For purposes of evaluation, the leverets were divided into groups according to the individual years in which they were admitted, according to the causes of their admission, and according to the reasons for their removal from rescue centre records.

The causes for the admission of young hares were divided into the groups given in Table 1. The reasons leading to their leaving rescue centres are shown in Table 2. The method of their division into these categories is following the instructions for recording handicapped animals in the Czech Republic. Animals that were still treated at the end of the period monitored in our study were not included in the analysis.

**Table 1:** Reasons for the admission of brown hare leverets to rescue centres in the Czech Republic

Reason for admission	Characteristics	Example
Orphaned	Young that have lost their mother	A young hare found without its mother
Caught needlessly	A young hare taken to a rescue centre without reason	A healthy, well fed young hare without obvious injury
Bitten by another animal	Bite wound	A young hare with bite injuries or seen being attacked by e.g. a dog
Injuries	Injuries, not including bite injuries	Fractures, contusions, injuries caused by garden machinery, etc.
Road traffic	Young hare injured by a road vehicle	An injured young hare found by the road
Late-born young	Young that are not mature in the autumn	Young of a low age found late in the year
Infection	Young with signs of infection (including parasites)	Young showing signs of coughing, diarrhoea, etc.
Exhaustion, starvation	Young of low weight, dehydrated, with no signs of infection	Dehydrated, emaciated young
Falls into sumps, chimneys, etc.	Falls into places from which young hares cannot escape	Young hare found in a recess from which it cannot escape
Destroyed nests	Young found by a destroyed nest with no possible shelter	Young near a nest destroyed by agricultural machinery
Other causes	Causes other than those given above	Shot, entangled in a foreign object, etc.

**Table 2:** Outcomes for brown hare leverets in rescue centres in the Czech Republic

Outcome	Description
**Release**	Young that recovered and were released back into their natural habitat
**Returned to the nest or adopted**	Young returned to the original nest they were taken from or added to hares that could care for them
**Death**	Young that died in rescue centres (unassisted death)
**Euthanasia**	Young euthanized by a veterinarian for health reasons
**Permanent captivity**	Young that had to remain in captivity due to permanent disability
**Escape**	Young that escaped during rehabilitation
**Unknown**	Not specified

The data were evaluated by the statistical program UNISTAT 6.5 for Excel (Unistat Ltd., London, UK). Spearman’s coefficient, which enabled the determination of a rank correlation coefficient, was used to evaluate the developmental trend of the figures in the years 2010 to 2019. A Chi-square test with Yates’ correction using the 2 × 2 contingency table method was used for the evaluation of differences in frequencies in individual groups with numbers of individuals > 5. A value of < 0.05 was stipulated as statistically significant in the statistical tests used.

## Results

**Fig. 1:**
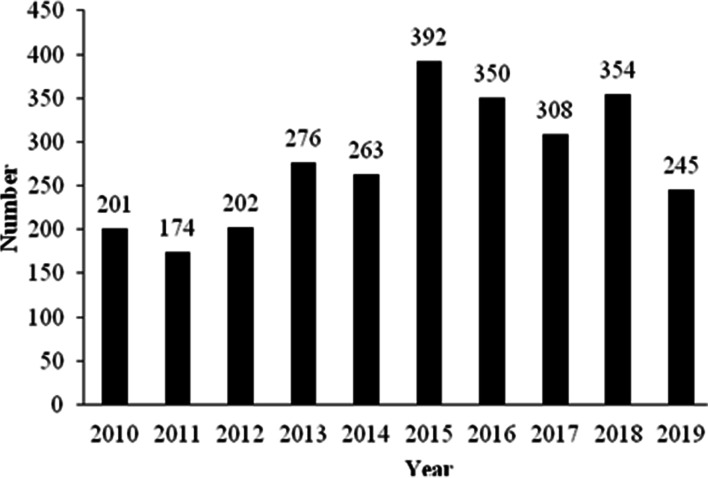
Number of brown hare leverets admitted to rescue centres in the period 2010–2019

A total of 2765 brown hare leverets were admitted to 34 rescue centres in the Czech Republic in the period from 2010 to 2019. We found an upwards trend in the number of young brown hares admitted to rescue centres in the Czech Republic during the studied period (rSp = 0.6364, *p* < 0.05). Figure 1 depicts the number of brown hare leverets admitted to rescue centres in the Czech Republic in the individual years in the studied period.

Reasons for the admission of brown hare leverets to rescue centres from 2010 to 2019 are shown in Table 3. The most frequent reason for the admission of brown hare leverets to rescue centres was the admission of orphaned young (49.15%) followed by the admission of young hares caught needlessly (19.60%) and young hares bitten by another animal (18.63%).

**Table 3:** Number and percentage of the total number of brown hare leverets admitted to rescue centres in the Czech Republic in the years 2010 to 2019 by reasons for admission

Reason for admission	young admitted (***n*** = 2765)	
	number	%
Orphaned	1359	49.15^a^
Caught needlessly	542	19.60^b^
Bitten by another animal	515	18.63^c^
Injured	78	2.82^e^
Road traffic	34	1.23^f^
Late-born young	25	0.90^f,g^
Infection	3	0.11^i^
Exhaustion, starvation	13	0.47^g,h^
Falls into sumps, chimneys, etc.	8	0.29^h,i^
Destroyed nests	151	5.46^d^
Other causes	37	1.34^f^

^a-i^ different superscript letters indicate a statistically significant difference between percentages in the column (*p* < 0.05)

The outcomes for young brown hares in rescue centres are given in Table 4. The most frequent outcome for brown hare leverets at rescue centres was death (40.76%), followed by release back into the wild (32.40%). A substantial proportion of young hares (19.58%) had to remain in permanent captivity after leaving the rescue centre. Euthanasia occurred in 3.29% of cases.

**Table 4:** Number and percentage of the total number of brown hare leverets admitted to rescue centres in the period 2010 to 2019 according to the outcomes

Outcome	number of young (*n* = 2630)	
	number	%
Release	852	32.40^b^
Returned to the nest or adopted	42	1.60^e^
Death	1072	40.76^a^
Euthanasia	103	3.29^d^
Permanent captivity	515	19.58^c^
Escape	9	0.34^f^
Unknown	37	1.41^e^

^a-f^ different superscript letters indicate a statistically significant difference between percentages in the column (p < 0.05)

The division of brown hare leverets into groups by the reason for their admission to rescue centres and the number of leverets released and those that died or were euthanized is given in Table 5. Brown hare leverets were released back into the wild most often in the case of admitted late-born young (52.00%) and those admitted after falls into sumps or chimneys (50.00%). The success rate was around a third in the case of orphaned young (34.51%), needlessly caught young (38.56%) and young admitted due to infectious diseases (33.33%). Death and euthanasia occurred most frequently in young hares following collisions with road vehicles (97.06%), bites by other animals (65.05%), injuries (70.51%) and animals suffering from exhaustion and starvation (76.92%). However, also 36.06% of orphaned young and 30.44% of needlessly captured young died or were euthanized.

**Table 5:** The division of brown hare leverets into groups by the reason for their admission to rescue centres and the number of leverets released and those that died or were euthanized

Reason for admission	Number of admitted (n = 2765)	Outcome			
		Released into the wild		Euthanized or died	
		number	%	number	%
Orphaned	1359	469	**34.51**	490	**36.06**
Caught needlessly	542	209	**38.56**	165	**30.44**
Bitten by another animal	515	94	**18.25**	335	**65.05**
Injures	78	16	**20.51**	55	**70.51**
Road traffic	34	1	**2.94**	33	**97.06**
Late-born young	25	13	**52.00**	12	**48.00**
Infection	3	1	**33.33**	2	**66.67**
Exhaustion, starvation	13	1	**7.69**	10	**76.92**
Falls into sumps, chimneys, etc.	8	4	**50.00**	1	**12.50**
Destroyed nests	151	37	**24.50**	63	**41.72**
Other causes	37	7	**18.92**	9	**24.32**

## Discussion

The number of brown hare leverets admitted to Czech wildlife rescue centres increased during the period 2010 to 2019. The finding that these were in many cases orphaned or needlessly captured young is particularly important. Even though the orphaned brown hare leverets and those needlessly captured are divided into two different groups, it is probable - because of the way of life of young hares - that at least some of the young believed to have been orphaned may have been needlessly captured. Young hares remain alone hidden in their nests, and their mother feeds them only once or twice a day, always in the same place and generally at the same time [19]. This survival strategy of young hiding quietly in their nests may arouse emotions in people who come across these young hares. They may then take them to rescue centres believing that they are orphaned, although these young do not, in reality, require help. It is far from easy to rear these young since the milk of hares has an extremely specific composition [20]. Substituting rabbit mothers for hare mothers is not entirely appropriate as the physiology of digestion and food intake in these two species show considerable differences [21]. Female hares also choose a special diet during lactation [22], and that is the reason why it is difficult to estimate correctly the ideal composition of milk. It is a problem mentioned by authors in a large number of wild animal species and the rearing of their young [23, 24]. The stress caused by a change of environment and by human handling also takes its toll on the health of young and may also harm attempts at their rearing [25]. This is also confirmed by the results of our study, in which only around a third of young hares admitted to rescue centres as orphaned or needlessly captured were successfully reared and released back into the wild, while around another third of these young animals died or were euthanized at wildlife rescue centres.

Bites by another animal were also a frequent reason for admission to wildlife rescue centres, as hare nests on the ground can easily be found both by domestic animals [26] and by natural predators, primarily foxes [27]. These animals could usually not be saved, and death or euthanasia occurred in 65% of cases. An even lower success rate was recorded in young hares admitted with other injuries or following a collision with a vehicle, for which the mortality rate amounted to 70 and 97%, respectively. The high mortality rate in the case of hares hit by a vehicle is not particularly surprising, as vehicles cause severe injuries to wild animals that are often incompatible with the treatment of any kind or any chance of survival, and are a frequent cause of the death of animals at rescue centres [28]. The frequency of these events may be an indirect index of hare population abundance.

Infection was listed as a reason for admission in only 3 animals. We suppose that only leverets with clinically obvious symptoms of infection (e.g. diarrhoea or respiratory symptoms, eye discharge, etc.) were listed in this category. Upon admission to the rescue centre, animals were not subjected to any specific diagnostic tests and thus, presence of infection in animals without clinical signs could not be detected. Alternatively, the leverets might have been brought to the rescue centres so young that no infection has yet occurred.

Many of the young hares taken to wildlife rescue centres were starved and emaciated and could not be saved, which may indicate that prompt intervention is important when young animals really do need help. In the case of hares, however, a correct assessment of the situation demands observation for a relatively long time, as mother hares visit their young extremely rarely over the course of any given day [19]. There is also the question of what causes the high percentage of animals that cannot be released back into the wild and that remain in permanent captivity (as was the case for 19.58% of individuals in our study) and what are the reasons for this when the animals taken to rescue centres are healthy. Such data are, however, not available in the rescue centre records. In the case of young animals reared in rescue centres and released back into the wild, it would also be appropriate to ascertain the rate of survival of these reared animals in the wild [13] and whether this factor, i.e. rearing in captivity, has any negative influence on their further survival in the wild.

There is a high mortality rate in young hares in the wild in the first weeks of life, previous studies reported leveret mortality ranging from 65% [9] to 84% [11] in the first month. Thus, it is particularly important to limit the factors that further contribute to their mortality. This involves, first and foremost, greater availability of suitable habitats, limiting losses on the roads and, perhaps most importantly, educating the public. Since orphaned leverets and leverets taken to rescue centres needlessly made up the largest proportion of young hares admitted to rescue centres in the Czech Republic, public education could help reduce these unnecessary interventions in the rearing of young whose chance of being released back into the wild is, according to our results, relatively low.

## Conclusion

Information on brown hare leverets admitted to wildlife rescue centres in the Czech Republic and the fate that awaits them reveal the principal risks to these animals in the wild and highlight the fact that the hare is a species subjected to a strong selection, both natural and artificial, as demonstrated by the difficulties it encounters in different environmental contexts, and especially those that are anthropized and/or subject to intensive agriculture. Public education would appear fundamental because of the large number of young animals that are probably taken to rescue centres entirely needlessly (including young declared as orphaned). Spreading awareness among the general public of how young hares are reared in the wild, where the absence of the mother in the nest need not be a sign of orphaned young, may prevent the unnecessary collection of young. It is far from easy to meet the specific demands of the rearing of leverets in captivity, and this leads to a low success rate even in the case of healthy young hares admitted to rescue centres. The chance of survival is minimal in the case of injured or otherwise weakened young, thus our efforts should be directed, first and foremost, at assuring conditions for rearing in the natural environment and protection against anthropogenic influences.

## Data Availability

Availability of data section of this manuscript in the Figshare repository DOI 10.6084/m9.figshare.14871975 in https://figshare.com/s/026fb318effd67aee1b8.
